# scProtoTransformer: Scalable reference mapping across molecules, cells, and donors

**DOI:** 10.1126/sciadv.aef0286

**Published:** 2026-08-28

**Authors:** Zhenchao Tang, Haohuai He, Shouzhi Chen, Jun Zhu, Tianxu Lv, Jiale Zhou, Jiehui Huang, Yaokun Li, Guanxing Chen, Linlin You, Calvin Yu-Chian Chen

**Affiliations:** ^1^ School of AI for Science, Peking University Shenzhen Graduate School, Shenzhen, China.; ^2^ School of Intelligent Systems Engineering, Shenzhen Campus of Sun Yat-sen University, Shenzhen, China.; ^3^ Department of Data Science and Artificial Intelligence, The Hong Kong Polytechnic University, Hong Kong SAR, China.; ^4^ Tsinghua-Peking Joint Center for Life Sciences, School of Life Sciences, Tsinghua University, Beijing, China.; ^5^ School of Artificial Intelligence and Computer Science, Jiangnan University, Wuxi, China.; ^6^ Medical Artificial Intelligence Laboratory, Westlake University, Hangzhou, China.; ^7^ Department of Computer Science and Engineering, The Hong Kong University of Science and Technology, Hong Kong SAR, China.; ^8^ Department of Computer Science, City University of Hong Kong (Dongguan), Dongguan, China.; ^9^ Department of Medical Research, China Medical University Hospital, Taichung, Taiwan.

## Abstract

The rapid accumulation of single-cell data has made it possible to comprehensively characterize biological systems at molecular, cellular, and donor levels. However, scalable reference mapping across different resolutions remains a major challenge in current research. Here, we propose scProtoTransformer, a prototype-based Transformer architecture designed to achieve scalable reference mapping across molecular, cell, and donor levels. scProtoTransformer introduces a knowledge-guided prototype tokenizer that projects gene expression into biologically interpretable pathway prototypes, effectively reducing numerical batch effects while preserving biological semantic patterns. Furthermore, by leveraging knowledge distilled from the foundation model and a dynamic supervised fine-tuning strategy, scProtoTransformer achieves robust biological representations with reduced pretraining requirements. Benchmark experiments across molecular, cell, and donor-level reference mapping demonstrate that scProtoTransformer delivers competitive or even superior performance compared with state-of-the-art approaches while providing interpretability through biological prototypes. Together, these results establish scProtoTransformer as a unified framework for scalable reference mapping, laying the foundation for systematic understanding from genes to individuals.

## INTRODUCTION

The rapid development of single-cell measurement and analysis technologies is driving a comprehensive understanding of biological systems at the gene, cell, and tissue levels. The rapid accumulation of data has already made deep learning the most mainstream solution in the field (*1*). Mapping annotations from reference datasets to query datasets remains a critical workflow in AI for Biology systems (*2*). Moreover, reference mapping based on atlases can further enable numerous downstream applications (*3*–*6*). Although many methods have been developed for reference mapping in cell identity annotation, scalable reference mapping across molecules, cells, and tissues remains an unexplored area. At the same time, multilevel representations are essential resources for building artificial intelligence–powered virtual cells (*7*).

However, achieving robust scalable reference mapping still faces two major challenges. The first is the classic problem: Gene expression, as a numerical measurement, is highly susceptible to batch effects (*8*–*10*). Differences in experimental platforms and sequencing depth can cause even the same cell type to exhibit notable discrepancies in expression profiles across datasets. Such “numerical drift” often obscures the true biological signals, leading models to learn technical noise rather than biological heterogeneity, thereby reducing generalization across datasets, platforms, and even laboratories. Thus, how to learn biologically meaningful cellular representations while avoiding interference from batch effects has long been a central challenge in single-cell analysis (*11*). A recent work on contrastive cell embedding learning with domain adaptation (*12*) has highlighted the importance of addressing batch effects through learned representations. Many current integration methods rely on predefined batch information for computation, but in real-world scenarios, the sources of batch effects are often unclear, and subjective batch definitions may severely compromise the robustness of research conclusions (*13*).

The second challenge lies in the current focus of existing methods, which is limited to cell-level reference mapping. The most popular approaches use pretrained foundation models to obtain cell representations and then use supervised fine-tuning (SFT) to transfer labels from reference data to query data (*14*–*18*). However, reference mapping across molecules, cells, and tissues has not yet been explored. Biological systems are inherently hierarchical (*19*): At the molecular level, gene-gene interactions influence biological functions such as pathways; at the cellular level, different gene set patterns describe the heterogeneity among individual cells; at the donor level, phenotypes constructed by groups of cells capture interindividual differences. Recent advances in multiomics integration and cancer subtype identification (*20*, *21*) have further demonstrated the importance of capturing biological heterogeneity across different resolutions. To date, there is still no multilevel framework that can achieve representation and reference mapping simultaneously at the molecular, cellular, and donor levels (*22*). We envision that such a framework would not only capture gene context at the molecular level and support cross-dataset integration and annotation at the cellular level but also generalize cell group characteristics at the donor level, thereby enabling a systematic understanding from molecules to individuals.

To address these two challenges, we propose scProtoTransformer, a prototype-based Transformer architecture for scalable reference mapping across molecular, cellular, and donor levels. scProtoTransformer does not require subjective batch selection; instead, it abstracts gene expression into a set of biological prototypes, thereby mitigating the impact of numerical batch effects while retaining biological semantics. Specifically, inspired by prototype-based modeling in computer vision (*23*–*25*), we introduce the prototype tokenizer, which linearly projects gene expression vectors into a prototype space. The projection matrix of the prototype tokenizer is initialized with biological priors, ensuring that each prototype corresponds to a biologically well-defined pathway. These prototypes are then treated as tokens and fed into stacked Transformer layers, where the self-attention mechanism models interactions among prototypes, capturing cross-functional biological associations while avoiding interference from raw expression values.

In addition, we design a knowledge distillation (KD) strategy guided by foundation models for scProtoTransformer. The goal is to enable scProtoTransformer to inherit the knowledge obtained by foundation models at great training cost, without requiring large-scale pretraining itself. Specifically, we use a frozen foundation model (e.g., scGPT) as a navigator and use its cell embeddings to supervise scProtoTransformer’s learning. Unlike the unsupervised pretraining strategy used by foundation models, our approach can dynamically leverage available labels: When labels are present, their discriminative power enhances scProtoTransformer’s biological representation capacity. To further train the model on limited data, we use dynamic SFT, which adaptively controls gradients to prevent overfitting.

Benchmark experiments demonstrate that scProtoTransformer supports multilevel reference mapping: The prototype tokenizer enables molecular-level reference mapping through gene embeddings, cell embeddings can be used for cell-level reference mapping, and aggregating embeddings of all cells from the same donor sample into existing multi-instance learning (MIL) modules allows for donor-level reference mapping. In summary, scProtoTransformer lays the foundation for integrative analysis across molecular, cellular, and donor levels.

## RESULTS

### scProtoTransformer overview

**Fig. 1.**
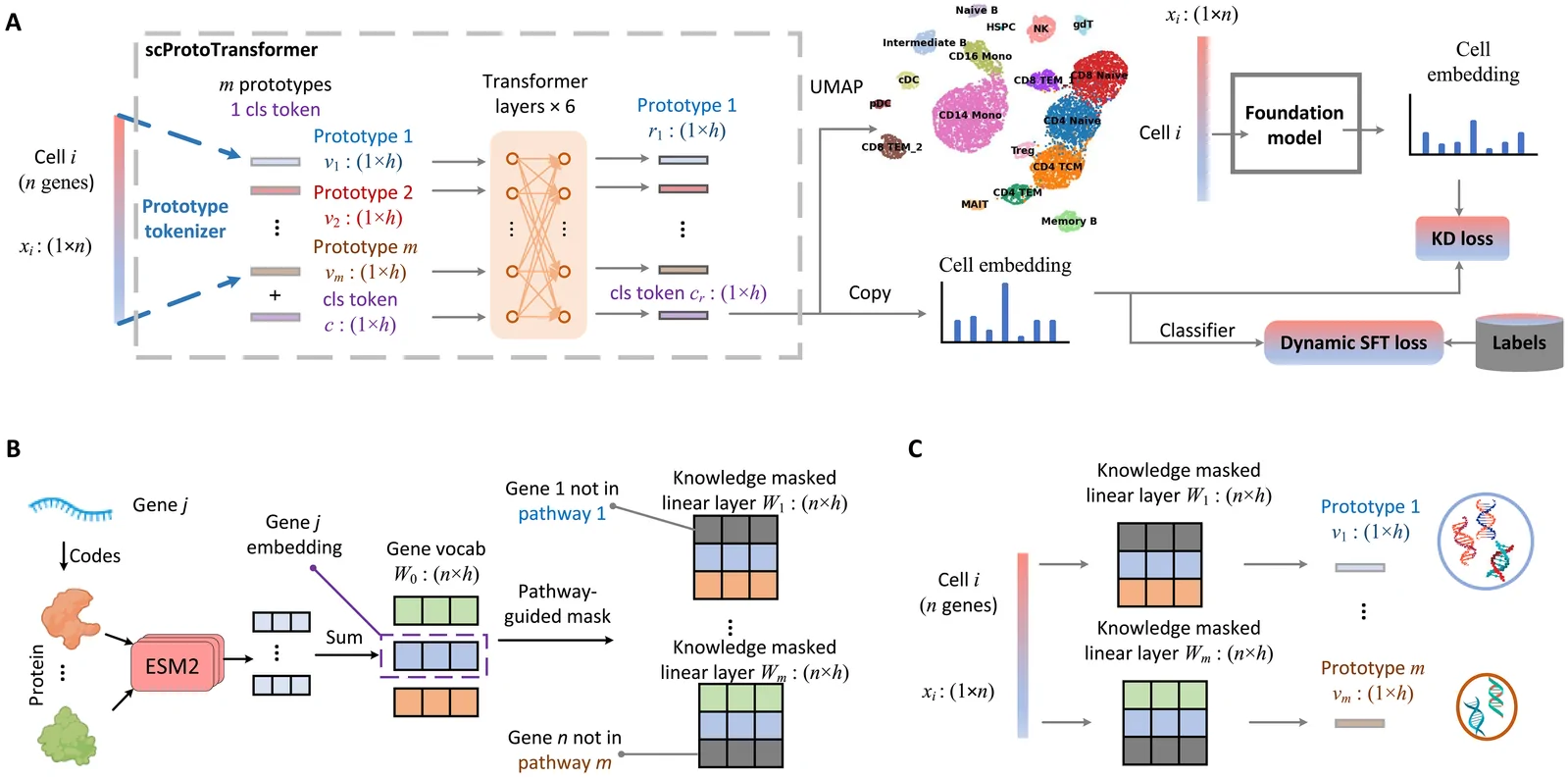
Architecture of scProtoTransformer. (**A**) For a given single cell, the gene space is transformed into prototypes that represent biological functions. The self-attention mechanism learns the interactions among prototypes to model the associations between biological functions. During pretraining, we distill scProtoTransformer from a frozen foundation model. Instead of cross-entropy, we use a dynamic SFT loss that adaptively controls gradients to prevent model overfitting. (**B**) Parameter initialization process of the prototype tokenizer. (**C**) The prototype tokenizer converts the gene expression profile of a single cell into multiple parallel prototype representations.

The workflow of scProtoTransformer is illustrated in Fig. 1A. For each input single cell, its gene expression profile is converted by the prototype tokenizer into multiple prototype tokens. Stacked Transformer layers then learn the interactions among these prototype tokens, modeling the associations between biological functions. The [CLS] token from the final Transformer layer is used as the cell embedding. In addition, we distill scProtoTransformer from the foundation model scGPT, requiring pretraining on only a small subset of scBaseCamp (*26*) (2 million cells sampled from 100 million, reducing the pretraining scale to 2% of the full dataset). Unlike unsupervised pretraining of foundation models, when label information is available in the data, we enhance the biological representation capacity of scProtoTransformer through Dynamic SFT.

More specifically, scProtoTransformer incorporates knowledge-based parameter initialization in the prototype tokenizer. As shown in Fig. 1B, we first construct a gene vocab, where each row represents a gene embedding. Each gene embedding is obtained by summing the embeddings of proteins encoded by the corresponding gene. Next, we apply a pathway-guided mask to transform the gene vocab into *m* parallel linear layers. As shown in Fig. 1C, these parallel linear layers convert gene expression into *m* prototype tokens.

After pretraining, each row in the gene vocab, i.e., the gene embeddings, supports molecular-level reference mapping. The cell embeddings produced by scProtoTransformer are used for cell-level reference mapping. For donor-level reference mapping, all cell embeddings from the same donor sample are aggregated through an MIL module to obtain the donor embedding.

### Molecular-level reference mapping

**Fig. 2.**
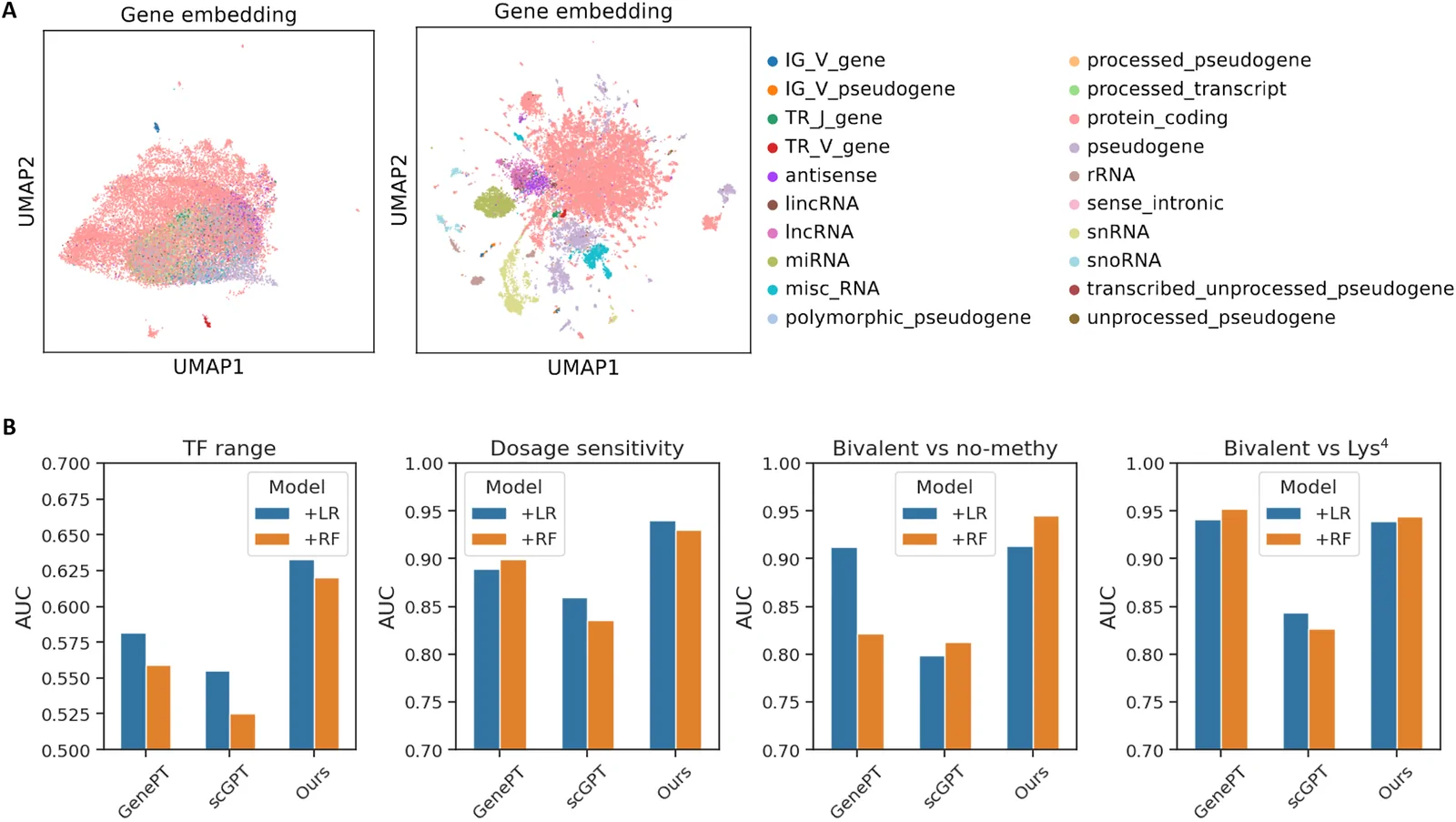
Molecular-level reference mapping. (**A**) UMAP visualization of the human whole genome. The left shows gene embeddings from the foundation model, whereas the right shows our gene embeddings, colored by gene functional categories. (**B**) Comparison of different methods across four molecular-level reference mapping tasks.

We extracted the gene vocabulary from scProtoTransformer and visualized the embeddings of 30,000 human genes using Uniform manifold approximation and projection (UMAP) (*27*). For comparison, we also extracted the gene embeddings from the foundation model scGPT. Using the labels provided in GenePT, we annotated each gene with its functional category. As shown in Fig. 2A, the UMAP visualization colored by gene function types reveals distinct clusters, indicating that scProtoTransformer encodes gene functions more clearly than the foundation model while ensuring that the gene embeddings preserve key biological information.

For gene-level reference mapping, we evaluated performance on specific biological tasks related to gene network dynamics. These tasks were drawn from datasets curated in the Geneformer benchmark (*14*): long-range versus short-range transcription factors (TFs), dosage-sensitive versus dosage-insensitive TFs, bivalent versus nonmethylated genes, and Lys^4^-only-methylated versus nonmethylated genes. For all four datasets, reference and query sets were partitioned according to the Geneformer protocol.

We compared our method with two mainstream approaches: GenePT (*28*) and scGPT (*17*). For each method, we performed reference mapping using logistic regression (LR) and random forest (RF) classifiers trained on the corresponding gene embeddings. Performance was evaluated using the area under the receiver operating characteristic curve (AUROC). As shown in Fig. 2B, our method consistently achieves competitive results, despite GenePT and scGPT benefiting from large language models and large-scale pretraining, respectively. These results highlight the potential of scProtoTransformer for molecular-level representation.

### Cell-level reference mapping

Given a single-cell input, scProtoTransformer can output a cell embedding. We evaluated cell-level reference mapping on three cell type annotation datasets. Each dataset has distinct characteristics: The bone marrow–derived mast cell (BMMC) dataset (*29*) is measured with single-cell RNA sequencing (scRNA-seq) and contains 13 batches and 22 cell types. The peripheral blood mononuclear cell (PBMC) dataset (*30*) is measured with single-cell assay for transposase-accessible chromatin sequencing (scATAC-seq) and contains two batches and six cell types. Following Muse-GNN (*31*), we used Seurat to convert scATAC-seq peaks into scRNA-seq genes. The Pan-cancer dataset (*32*, *33*) is measured with scRNA-seq but involves heterogeneous cell types across multiple cancers, making annotation more challenging. Pan-cancer includes 13 cancer types and 23 cell types, with each cancer treated as a separate batch.

Data preprocessing followed the standard pipeline of scanpy. For BMMC, we selected the first six batches as the reference dataset and the remaining seven batches as the query dataset. For PBMC, we used the first batch as the reference dataset and the remaining batch as the query dataset. Pan-cancer was used to validate scProtoTransformer’s performance in cross-disease cell type annotation. Specifically, three cancers [ESCA (esophageal carcinoma), THCA (thyroid carcinoma), and UCEC (uterine corpus endometrial carcinoma)] were used as the reference dataset, whereas the other cancers (batches) served as the query dataset.

**Fig. 3.**
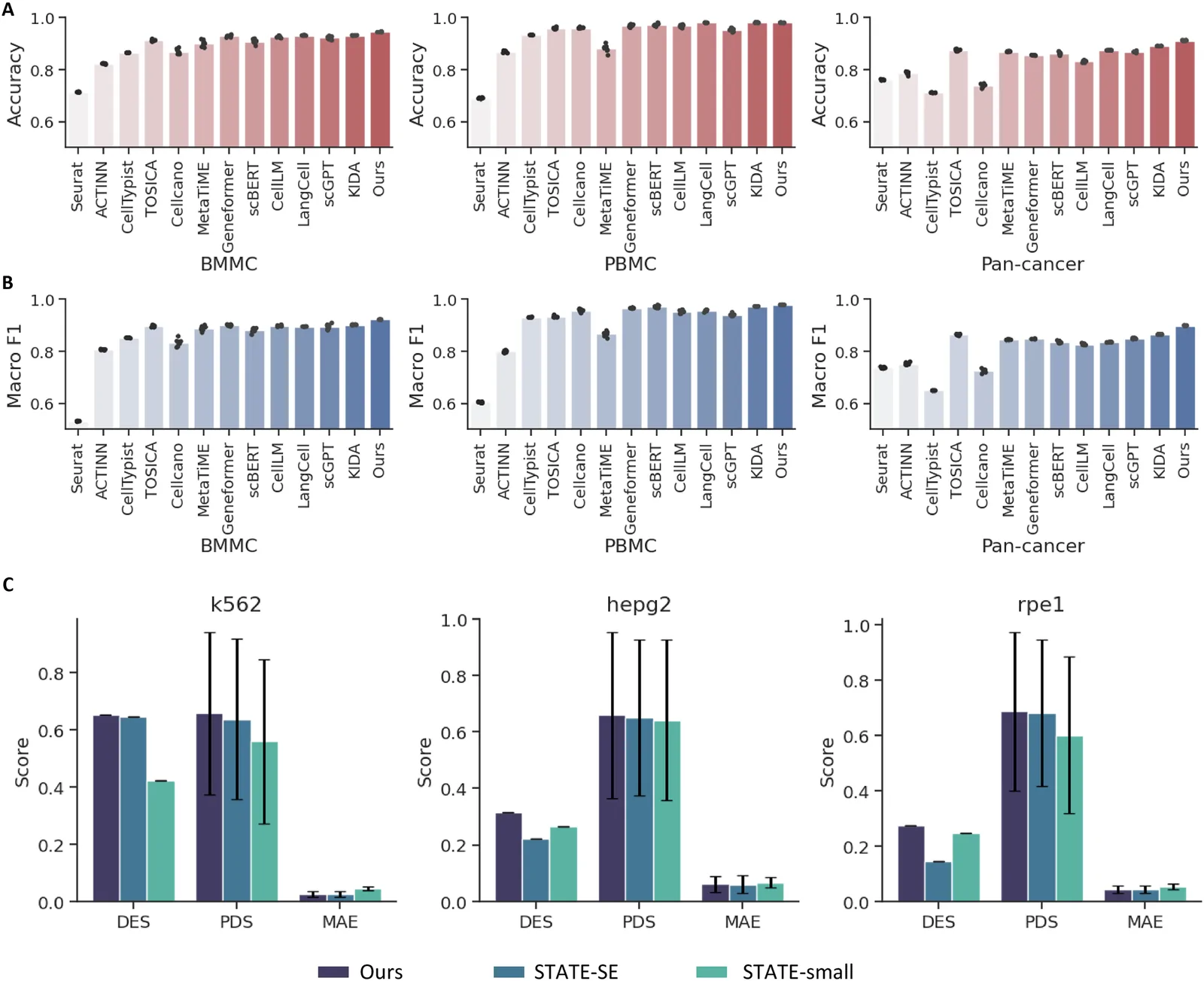
Cell-level reference mapping. (**A**) Comparison of Acc across different methods on cell-level reference mapping tasks. (**B**) Comparison of F1 scores across different methods on cell-level reference mapping tasks. (**C**) Performance comparison on single-cell perturbation response prediction.

We compared our method against the following annotation baselines: Seurat (*34*), CellTypist (*35*), ACTINN (*36*), TOSICA (*37*), Cellcano (*38*), MetaTiME (*39*), Geneformer (*14*), scBERT (*15*), CellLM (*40*), LangCell (*41*), scGPT (*17*), and KIDA (*42*). For cell type annotation, we used accuracy (Acc) and F1 score as evaluation metrics. As shown in Fig. 3 (A and B), our method achieved superior cell type annotation results. Although foundation model–based methods generally performed well, their maintenance and data management are highly complex. Small models such as KIDA and TOSICA also showed strong performance, but they require retraining from scratch on each dataset, which poses a challenge for rapid deployment (training from scratch requires more complex hyperparameter design). By contrast, our method requires neither maintaining a complex foundation model corpus nor retraining from scratch while still achieving competitive performance on cell-level reference mapping tasks.

For the two different modalities (BMMC: scRNA-seq; PBMC: scATAC-seq), we visualized the cell embeddings using UMAP. As shown in fig. S1, compared with modality-specific methods and the original principal components analysis (PCA), the cell embeddings generated by scProtoTransformer not only eliminated batch effects but also enhanced biological heterogeneity (the discriminability between cell types).

Furthermore, within the research perspective of virtual cells (*7*), our cell-level embeddings also facilitate tasks involving the perturbation response prediction. We obtained cell embeddings from scProtoTransformer and then used these cell embeddings as the input for the STATE (*43*) decoder. As shown in Fig. 3C, this yields zero-shot performance comparable to the standard STATE model across three datasets (*44*). This result suggests that scProtoTransformer can provide a competitive cell representation without the full perturbation-specific pretraining.

### Explainability of prototypes

Cell-level embeddings exhibit strong biological heterogeneity, which can be analyzed through prototypes. scProtoTransformer provides inherent interpretability because each prototype can be named by a specific pathway. The input to each Transformer layer consists of a [CLS] token, representing the cell embedding, along with a set of prototype tokens. Thus, each cell embedding can extract attention values associated with all pathways. By aggregating across all cells, we obtain an attention expression matrix. We then perform differential analysis on this matrix—for example, selecting the top 6 CD8+ T cell–specific pathways and visualizing them with a heatmap, as shown in fig. S2A. Taking the top-ranked pathway as an example, we retained the genes involved in this pathway and examined their expression distribution across different cell types. We found that these genes are highly expressed in CD8+ T cells (fig. S2B), indicating that the attention learned by scProtoTransformer is consistent with the observed biological distribution, thereby demonstrating the interpretability of its prototype-based design.

Building on our previous strategy in KIDA for selecting top-ranked genes, we retained cell type–specific genes and constructed a gene coexpression network based on their embeddings. As shown in fig. S3A, we first obtained 20 CD8+ T cell–specific gene embeddings. By calculating similarities among these embeddings, we generated a graph representing the CD8+ T cell–specific coexpression network. We identified five genes with particularly strong similarity. Visualizing their expression patterns with UMAP (fig. S3B) confirmed that these genes are highly expressed in the CD8+ T cell region. Notably, when compared with CellMarker (*45*), all of these genes are known marker genes for CD8+ T cells.

### Cross-modal and cross-batch integration

Because most downstream tasks benefit from a unified cell-level atlas, we further validated the representation capability of scProtoTransformer through integration tasks. We first evaluated multimodal integration. We adopted the benchmark we previously established in Monae (*46*) and selected three challenging unpaired multimodal datasets: 10x Multiome (*47*): jointly profiled 9631 human PBMCs using 10x Multiome, with the pairing information between RNA and ATAC modalities removed; Ma2020 (*48*): profiled 32,231 mouse skin cells with Simultaneous High-throughput ATAC and RNA Expression with sequencing (SHARE-seq) to obtain RNA+ATAC modalities, with pairing information removed; and Muto2021 (*49*): profiled a total of 44,190 cells from the human kidney using snRNA-seq and snATAC-seq. The RNA modality contained 19,985 cells, and the ATAC modality contained 24,205 cells.

**Fig. 4.**
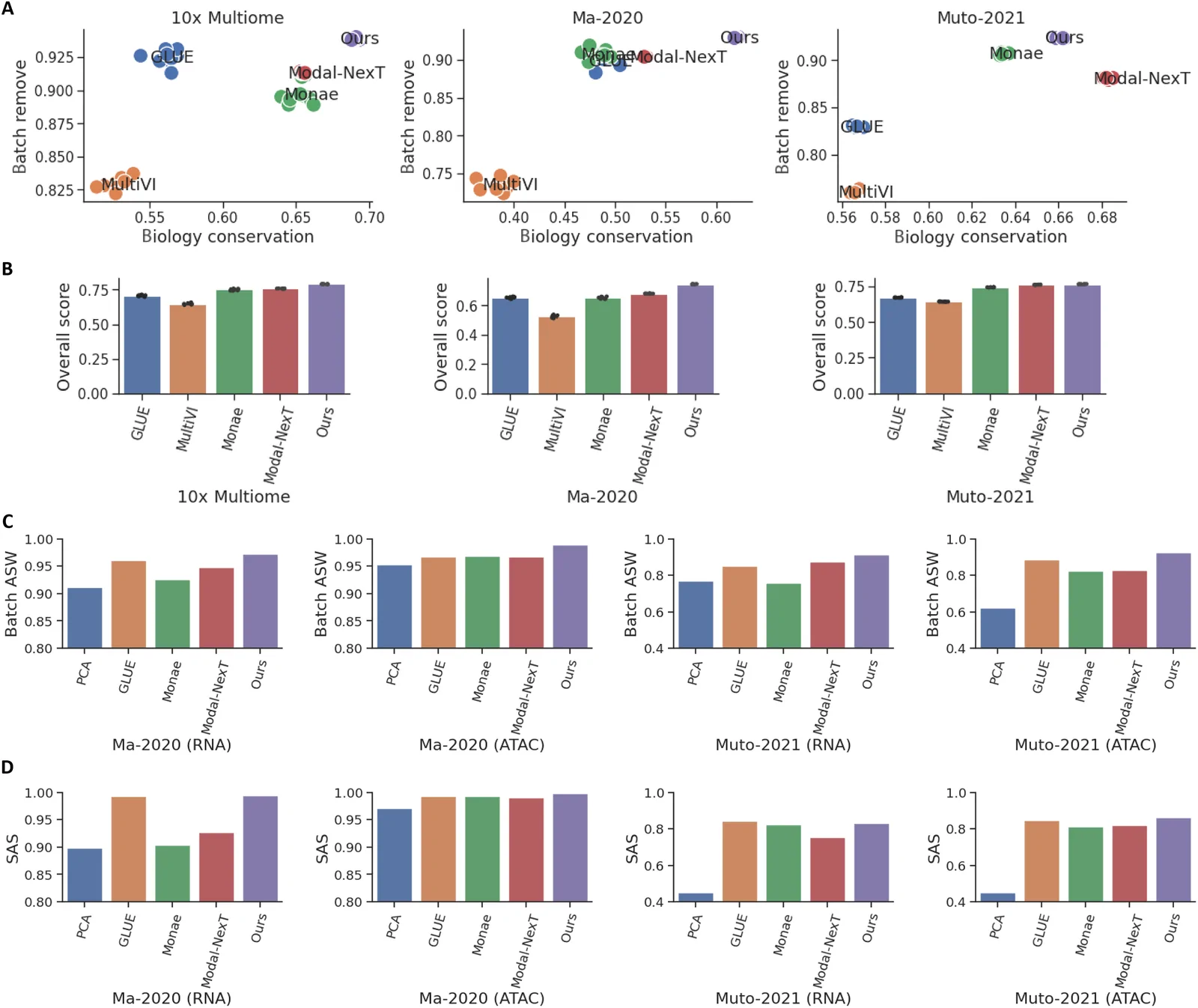
Comparison of multimodal integration results and batch integration results. (**A**) Simultaneous comparison of batch effect removal and biological heterogeneity preservation. (**B**) Overall score comparison of multimodal integration. (**C**) Comparison of batch ASW across different batch integration methods. (**D**) Comparison of SAS across different batch integration methods. In batch integration, PCA refers to cell embeddings without batch correction.

We compared scProtoTransformer against several specialized integration methods: GLUE (*47*), MultiVI (*50*), Monae (*46*), and Modal-NexT (*51*). We used three widely adopted evaluation metrics in multimodal integration: batch removal, biology conservation, and overall score. As shown in Fig. 4A, scProtoTransformer achieved the best balance between batch effect removal and biological conservation. As shown in Fig. 4B, scProtoTransformer outperformed state-of-the-art integration-specific methods in terms of overall score.

For Ma2020 and Muto2021, each modality consisted of four to five batches, enabling evaluation of batch integration metrics within each modality. The batch integration metrics include batch ASW (average silhouette width) and SAS (Seurat alignment score). As shown in Fig. 4 (C and D), scProtoTransformer was able to automatically achieve batch integration during multimodal integration, without requiring adversarial training strategies as in GLUE.

### scProtoTransformer is suitable for spatial data

In multicellular organisms, cells within a tissue slice interact with each other and form spatial structural patterns, whereas batch effects are often more pronounced across different slices. Existing specialized spatial analysis methods typically require incorporating additional nonmolecular features (e.g., spatial coordinates) to learn a joint representation across slices, highlighting the limitations of scRNA-seq analysis methods in handling spatial data. In contrast, scProtoTransformer has an inherent ability for batch integration, allowing it to be directly adapted to spatial data without relying on nonmolecular features such as spatial coordinates, which can introduce subjective biases. We validated the adaptability of scProtoTransformer on the dorsolateral prefrontal cortex (DLPFC) dataset (*52*), which contains normalized mRNA expression and two-dimensional (2D) spatial coordinates for each cell.

**Fig. 5.**
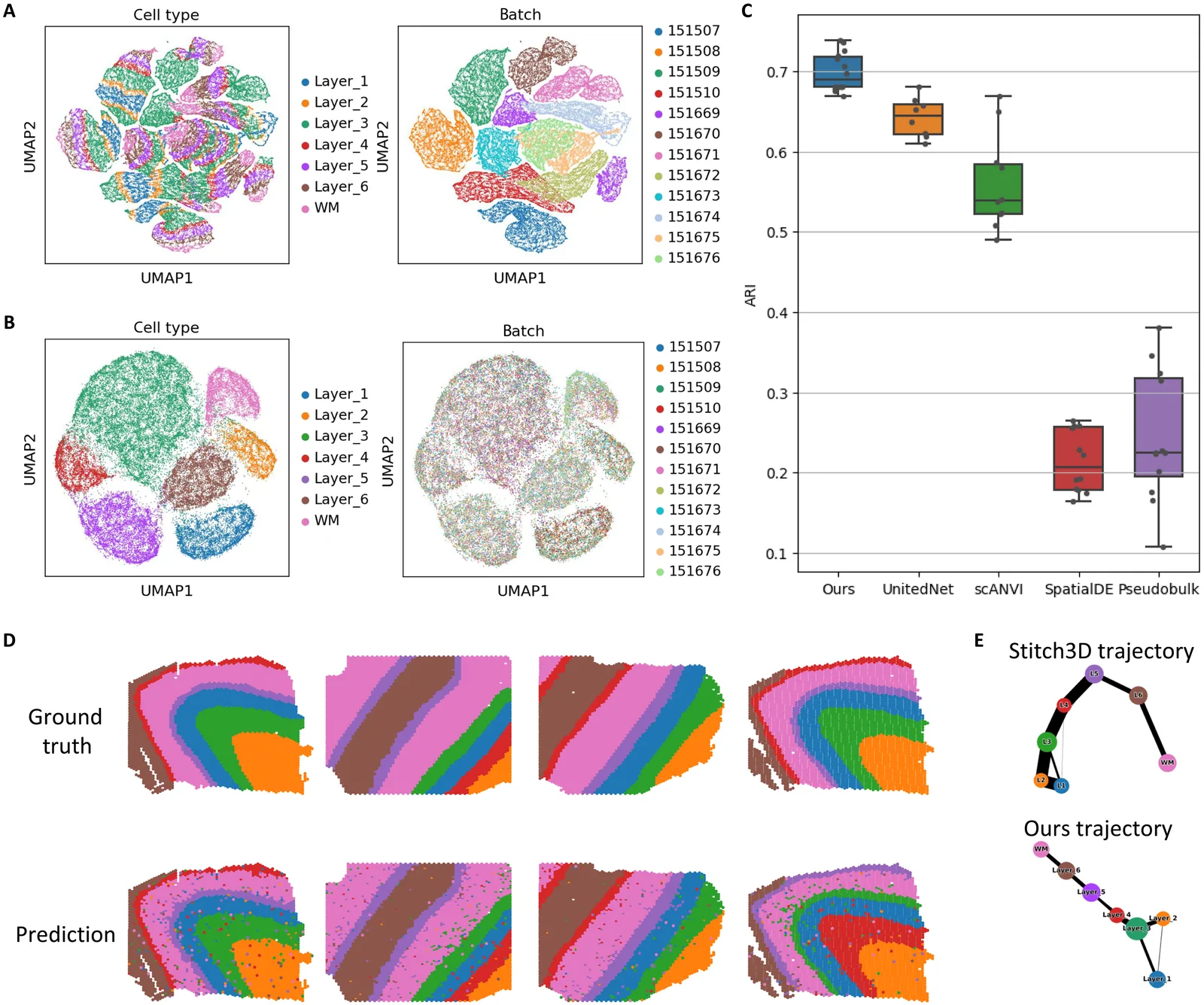
Applications on spatial data. (**A**) UMAP visualization of multislice data after direct dimensionality reduction, colored by cell type and batch label, respectively. (**B**) UMAP visualization of cell embeddings output by scProtoTransformer, colored by cell type and batch label, respectively. (**C**) Comparison of results of different spatial data representation methods; we use the ARI to measure the biological heterogeneity of cell embeddings. (**D**) Visualization on slices; we compare the actual cell types with the predicted cell types. (**E**) Comparison of trajectory inference results.

As shown in Fig. 5A, UMAP visualization of PCA embeddings across multiple slices revealed strong batch effects, with cell embeddings from different slices completely separated. By contrast, UMAP visualization of scProtoTransformer cell embeddings (Fig. 5B) showed complete mixing across slices, whereas clustering according to cell types could still be clearly observed. We compared scProtoTransformer with specialized spatial analysis methods [UnitedNet (*53*), scANVI (*54*), spatialDE (*55*), and Pseudobulk (*52*)] using the adjusted Rand index (ARI) as the evaluation metric, and as shown in Fig. 5C, our method achieved superior representation quality.

We further performed cell type reference mapping on the DLPFC dataset, which consists of 12 slices. We randomly selected eight slices as the reference dataset and used the remaining four slices as the query dataset. As shown in Fig. 5D, the predictions made by scProtoTransformer were highly consistent with the ground-truth annotations. Moreover, we compared our approach with Stitch3D (*56*), a more advanced 3D spatial transcriptomics method that leverages 3D spatial coordinates to accurately infer cell differentiation trajectories. Using scProtoTransformer’s cell embeddings combined with PAGA (*57*) for trajectory inference, we obtained consistent results (Fig. 5E).

### Donor-level reference mapping

Multiple studies have demonstrated that MIL can effectively bridge donors and single-cell representations (*58*–*60*). In MIL, a donor sample is treated as a bag, and the cells within the sample are treated as instances. Typically, the cancer phenotype of the donor sample (bag label) is known, whereas the phenotype of individual cells (instance labels) is unavailable. According to clinical diagnostic definitions, the relationship between bag and instance labels is as follows: If all instances in a bag are normal cells, the bag is defined as a normal bag; if a bag contains any tumor cells, it is defined as a tumor bag.

**Fig. 6.**
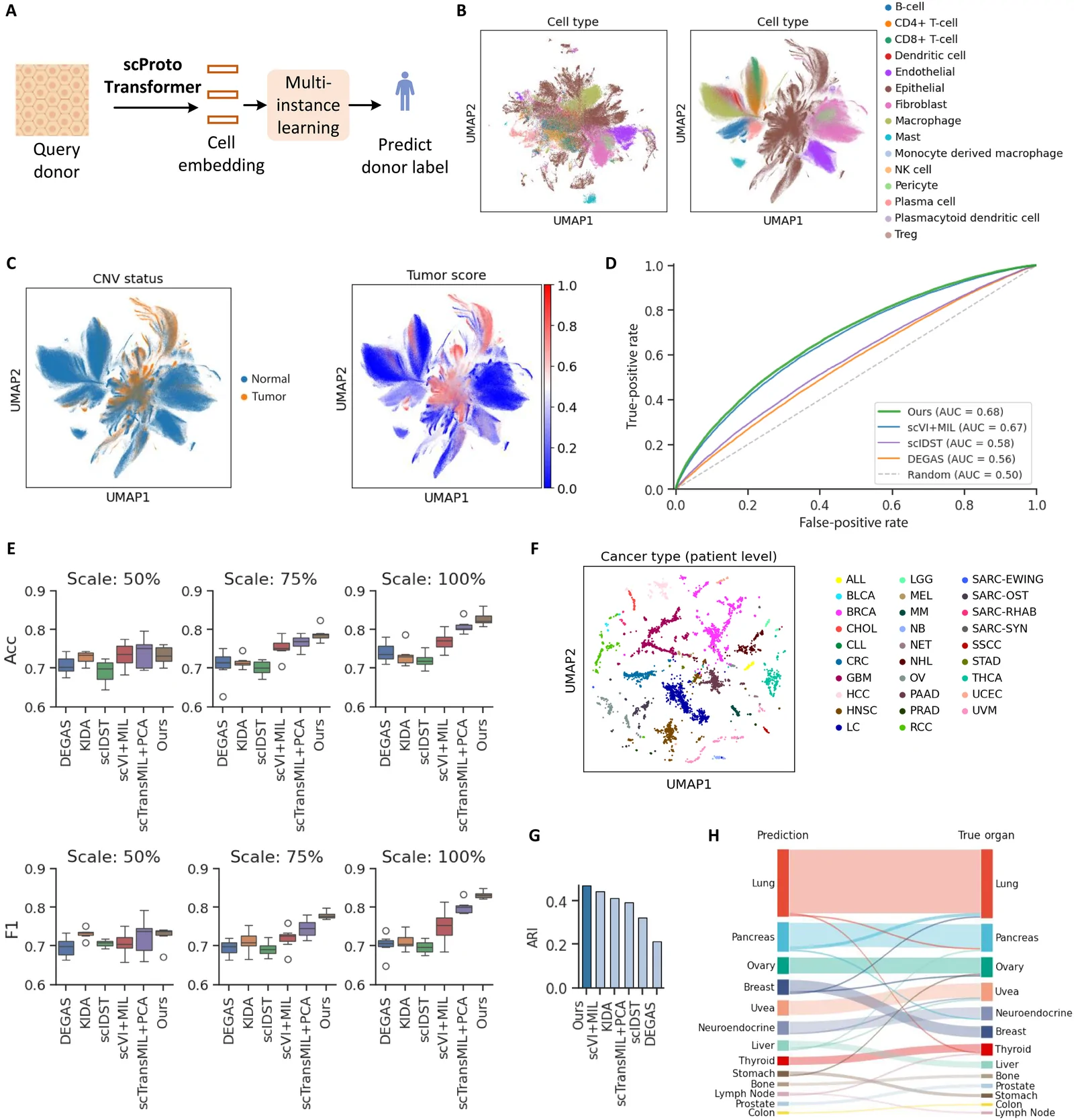
Donor-level reference mapping. (**A**) Workflow of donor-level reference mapping using scProtoTransformer. (**B**) Kang-2024 cell embeddings (PCA versus scProtoTransformer), colored by cell type. (**C**) Single-cell tumor labels (left) and inferred tumor scores (right), demonstrating donor–to–single-cell traceability. (**D**) Tumor prediction ROC performance comparison at single-cell resolution. (**E**) Comparison of donor-level reference mapping results under different scales of training data. (**F**) UMAP visualization at the donor level, colored by cancer type. (**G**) Comparison of embedding heterogeneity at the donor level. (**H**) Cancer type prediction results on metastatic samples.

For a donor sample, we first obtain cell embeddings from scProtoTransformer and then input them into the MIL module, which aggregates them into a donor embedding. We then use donor-level embeddings for reference mapping, as shown in Fig. 6A. By updating the MIL module based on the bag labels of reference donors, the module not only predicts the disease state of a donor but can also trace back—through the aggregation weights—which cells in the sample are malignant.

We demonstrate this on the Kang-2024 dataset (*61*) preprocessed in scTransMIL (*58*), which is composed of 104 publicly available cancer datasets. It covers 20,000 donor samples, 4 million single cells, and 30,000 genes. For reference mapping, we only use donor-level cancer labels (tumor or normal).

We visualized the PCA cell embeddings of Kang-2024 (colored by cell type) and the cell embeddings generated by scProtoTransformer, as shown in Fig. 6B. The comparison shows that clusters of different cell types are well separated, indicating that scProtoTransformer recovers the underlying biological heterogeneity.

In addition, we used the copy number variation (CNV) labels provided in Kang-2024 as ground truth at single-cell resolution (Fig. 6C, left). We then compared them with tumor scores at single-cell resolution inferred by the MIL module (Fig. 6C, right). The inferred results are consistent with the ground truth. Furthermore, to statistically evaluate the reliability of our cell-level predictions, we performed an ROC analysis (Fig. 6D) using CNV-based annotations as the primary benchmark. Our method demonstrated superior performance. These results indicate that scProtoTransformer achieves highly accurate cancer phenotype inference across a pan-cancer atlas encompassing 4 million single cells.

For donor-level evaluation, we used Acc and F1 score. The Kang-2024 dataset was randomly split into reference (80% of donors) and query (20% of donors) sets, ensuring no overlap of donors between the two, thereby directly assessing the model’s generalization to unseen donors. From the original reference set, we further sampled subsets of 50, 75, and 100% of donors. We trained on each subset and tested on the same query set. The performance of different methods [DEGAS (*62*), KIDA (*42*), scIDST (*60*), scVI+MIL (*59*), and scTransMIL (*58*)] is shown in Fig. 6E. As the size of the reference data increased, all methods improved, but the improvement of scProtoTransformer was more notable, outperforming the baselines. This highlights the potential of scProtoTransformer for cancer detection at the donor level. We also visualized donor-level embeddings with UMAP, colored by cancer type (Fig. 6F), showing that our method effectively captures donor-level phenotypic heterogeneity. We evaluated the donor sample embeddings generated by different methods using the ARI in conjunction with cancer types. Our method demonstrated the highest discriminative power, as illustrated in Fig. 6G.

Beyond achieving precise tumor/normal classification, scProtoTransformer also supports multiclass tasks, such as cancer type prediction. This holds particular clinical relevance and value for highly challenging applications like Cancer of Unknown Primary (CUP). CUP represents a major diagnostic dilemma: Although metastatic lesions are detected, the primary tumor cannot be identified. This diagnostic uncertainty poses a severe clinical challenge as accurately identifying the tumor’s origin is critical for determining optimal treatment strategies yet remains exceptionally difficult in these cases. Leveraging cancer type annotations from a pan-cancer dataset (comprising 30 different types), our method achieved impressive classification performance on metastatic tumor samples within an independent test set. As shown in Fig. 6H, the predicted cancer types align closely with the true tissue of origin. The robust performance demonstrated on metastatic specimens is particularly noteworthy as it addresses a critical and unmet clinical need in the diagnosis and management of CUP.

### Ablation study

scProtoTransformer benefits from three key components: the knowledge-based prototype tokenizer, the KD loss derived from the foundation model, and the dynamic SFT loss.

To systematically evaluate the contribution of each component, we conducted ablation experiments by individually removing the pathway-guided mask in the prototype tokenizer (w/o Proto), the KD loss (w/o KD), and replacing dynamic SFT with standard cross-entropy fine-tuning (w/o DFT) and then assessed performance changes across molecular-, cell-, and donor-level tasks.

As shown in Table 1, removing the mask from the prototype tokenizer caused the most notable performance drop at the molecular level. This result confirms the critical role of the prototype tokenizer in mapping raw gene expression into biologically meaningful pathway space. It effectively filters out numerical batch noise while preserving and amplifying functional semantics of genes. In contrast, removing the KD loss or dynamic SFT loss had only a minor effect at the molecular level.

**Table 1.** Ablation study results of scProtoTransformer across molecular, cell, and donor levels. “w/o Proto” removes the pathway-guided mask; “w/o KD” removes KD; “w/o DFT” replaces dynamic SFT with standard cross-entropy fine-tuning (i.e., standard SFT); “Random Init” replaces ESM2-based gene vocabulary initialization with random initialization while retaining the pathway-guided mask.

Level	Task	Full	w/o Proto	w/o KD	w/o DFT	Random Init
Molecule	TF range	0.62	0.55	0.60	0.59	0.56
	Dosage sensitivity	0.93	0.85	0.91	0.90	0.88
	Bivalent versus no-methy	0.95	0.82	0.90	0.92	0.91
	Bivalent versus Lys^4^	0.94	0.86	0.92	0.91	0.87
Cell	BMMC	0.94	0.92	0.88	0.85	0.91
	PBMC	0.97	0.94	0.91	0.87	0.96
	Pan-cancer	0.91	0.86	0.84	0.85	0.87
Donor	Kang-2024 (mean)	0.81	0.79	0.70	0.71	0.77
	Kang-2024 (attn)	0.83	0.82	0.76	0.72	0.75

At the cell and donor levels, however, removing the KD loss or replacing dynamic SFT with standard SFT (w/o DFT) resulted in marked performance degradation. This indicates that the KD loss transfers the generalization ability of the foundation model through KD, whereas the dynamic SFT loss enhances discriminability and robustness under limited labeled data by adaptively controlling gradients and preventing overfitting. This effect is further amplified in donor-level tasks. Notably, the advantage of dynamic SFT over standard SFT is most pronounced at the cell and donor levels, whereas the difference at the molecular level is marginal. This is because the stop-gradient scaling factor $\text{sg}\left[\pi_{\theta }\left(y^{*}|x\right)\right]$ in dynamic SFT effectively prevents gradient explosion when $\pi_{\theta }\left(y^{*}|x\right)$ is low, which is particularly beneficial in label-scarce scenarios such as donor-level tasks.

Furthermore, to isolate the contribution of ESM2 protein-level prior knowledge from the structural contribution of the pathway mask, we replaced ESM2 initialization with random initialization [Xavier (*63*)] while retaining the pathway-guided mask. As shown in the “Random Init” column of Table 1, random initialization led to performance degradation across all tasks, with a more pronounced effect at the molecular level. This demonstrates that ESM2 protein prior knowledge and the pathway mask structure provide complementary performance gains. Without ESM2 initialization, the pathway mask alone still yields notable improvement over complete mask removal (w/o Proto in Table 1), confirming the independent value of each component.

We also evaluated the sensitivity of the KD strategy to the choice of teacher model (Table 2). The frozen scGPT yields the best results. scFoundation (frozen) and Geneformer (frozen) also achieve strong performance with only marginal drops, demonstrating that the KD strategy generalizes across different foundation model architectures. In contrast, fine-tuning the teacher model jointly with scProtoTransformer consistently degrades performance. We attribute this to the fact that the data sampled from scBaseCamp for scProtoTransformer training is only a local subset, which disrupts the teacher’s pretrained knowledge when its parameters are unfrozen. Crucially, all teacher configurations outperform the no-KD baseline, demonstrating that the performance gain primarily stems from the foundation model–based KD strategy itself rather than the specific properties of a particular teacher model.

**Table 2.** Teacher model sensitivity analysis for KD. “frozen” denotes that the teacher parameters are fixed; “fine-tuning” denotes joint parameter update during training, which degrades performance because the locally sampled scBaseCamp subset disrupts the teacher’s pretrained knowledge.

Teacher model	BMMC	PBMC	Pan-cancer	Kang-2024 (mean)	Kang-2024 (attn)
scGPT (frozen)	0.94	0.97	0.91	0.81	0.83
scGPT (fine-tuning)	0.90	0.95	0.86	0.76	0.78
Geneformer (frozen)	0.91	0.94	0.93	0.82	0.81
Geneformer (fine-tuning)	0.89	0.92	0.85	0.75	0.77
scFoundation (frozen)	0.96	0.97	0.92	0.80	0.81
scFoundation (fine-tuning)	0.91	0.93	0.86	0.77	0.79
w/o KD (no teacher)	0.88	0.91	0.84	0.75	0.76

Overall, the ablation study validates the necessity of all three modules in scProtoTransformer: The prototype tokenizer drives molecular-level semantic modeling, whereas the KD loss and dynamic SFT loss jointly optimize discriminative representations at the cell and donor levels. Together, these components support cross-scale reference mapping.

## DISCUSSION

Single-cell omics analysis is entering the era of foundation models capable of integrating knowledge across diverse datasets. Despite notable progress made by large-scale foundation models, two major bottlenecks remain: persistent batch effects and the absence of a scalable, multilevel representation framework spanning molecular, cellular, and donor resolutions. We address these challenges with scProtoTransformer, a prototype-based Transformer that provides a unified, efficient, and interpretable solution for cross-scale reference mapping.

Instead of relying on raw expression values that are inherently noisy and sensitive to numerical variation, scProtoTransformer abstracts these signals into a set of stable functional prototypes defined by biological priors (pathways). This transformation effectively acts as a biological normalization layer, mitigating batch-induced “numerical drift” while preserving biologically meaningful patterns. The subsequent Transformer layers model interactions between these functional prototypes, producing cell embeddings that exhibit enhanced biological heterogeneity and reduced technical variation, as validated by our performance on multimodal and batch integration tasks.

In addition, the stable training of scProtoTransformer benefits from our KD strategy. By leveraging frozen large-scale foundation models (e.g., scGPT) as teachers, we distill high-quality representations into scProtoTransformer, whereas dynamic SFT ensures biologically consistent gradient updates when labels are available. This allows scProtoTransformer to inherit the representational power of foundation models without requiring resource-intensive pretraining, making it easier for researchers to adapt foundation model knowledge into customized models.

scProtoTransformer systematically unifies representation learning across molecular, cell, and donor levels. Learned gene embeddings naturally cluster by function, enabling high-quality molecular-level reference mapping. At the cellular level, scProtoTransformer builds joint cell representations that facilitate integration and annotation, with prototype attention providing intrinsic interpretability by linking cell identities to specific active pathways. At the donor level, aggregating cell embeddings yields donor representations suitable for patient stratification and disease monitoring, whereas the aggregation weights allow inference of malignant or disease-relevant cell populations within a donor.

Comprehensive benchmarks across multiple levels demonstrate that scProtoTransformer has the potential to serve as a unified framework for scalable reference mapping, thereby laying the foundation for systematic understanding of biological systems from genes to individuals.

## MATERIALS AND METHODS

### Prototype tokenizer

The prototype tokenizer initializes model parameters with biological priors guided by known biological pathways. This initialization ensures that the learned prototypes correspond to biologically meaningful representations rather than purely numerical patterns.

The gene embeddings are derived from the protein language model ESM2 (*64*). ESM2 is a transformer-based protein language model pretrained on ∼65 million protein sequences from UniRef50 using masked language modeling. We use the ESM2-650M variant (33 layers, 1280 embedding dimension, and 650 million parameters), which outputs a per-residue embedding of dimension *h* = 1280. Given a protein amino acid sequence, we obtain the protein-level embedding $E_{p}\in {\mathbb{R}}^{h}$ by mean-pooling over all residue embeddings.

The process begins by constructing a gene vocabulary (Gene Vocab) $W_{0}\in {\mathbb{R}}^{n\times h}$, where each row represents the embedding of a gene. The embedding $E_{g}\in {\mathbb{R}}^{h}$ of each gene *g* is computed by summing the embeddings of the proteins encoded by the gene

$$E_{g}=\sum_{p\in P_{g}}E_{p}$$

where $P_{g}$ is the set of proteins associated with gene *g*, and $E_{p}\in {\mathbb{R}}^{h}$ is the embedding for protein *p* from ESM2 (*64*). These gene embeddings form the initial gene vocabulary $W_{0}$, providing biologically informed initialization that encodes protein-level structural and functional information into the prototype tokenizer.

Next, a pathway-guided mask is applied to the Gene Vocab. This mask, based on known biological pathways, ensures that the model focuses attention on biologically relevant gene sets. The mask transforms the Gene Vocab into multiple parallel linear weights, each of which projects the gene expression into a prototype space.

For the knowledge-based mask, we create a list consisting of all gene names in reference dataset and obtain *m* pathways corresponding to this gene list through gene set enrichment analysis (GSEA) combined with pathway annotation (gmt file on the web or locally). We add mask to Gene Vocab (a linear matrix) according to the relationship between genes and pathways. Assuming that gene 1 is not in pathway 1, the first row (gene 1) of linear matrix $W_{1}\in {\mathbb{R}}^{n\times h}$ (pathway 1) is all masked to value 0. Conversely, assuming that gene *n* is in pathway 1, the *n*th row of linear matrix $W_{1}\in {\mathbb{R}}^{n\times h}$ will not have a mask added. Last, we get *m* matrices with masks (a parallel linear layer).

The parallel linear layer projects gene expression data into *m* prototypes, with each prototype token representing a specific biological pathway or function. The transformation from gene expression $x_{i}\in {\mathbb{R}}^{1\times n}$ (a row vector) for a given cell *i* to prototype tokens $v_{p}\in {\mathbb{R}}^{1\times h}$ is performed by applying the masked matrix of prototype *p*

$$v_{p}=x_{i}W_{p}$$

where $W_{p}\in {\mathbb{R}}^{n\times h}$ is a masked linear weight matrix for prototype *p*, with *n* being the number of genes and *h* being the embedding dimension. This formulation is consistent with Fig. 1C, where the row vector $x_{i}$ is right multiplied by the weight matrix $W_{p}$ to produce the prototype token $v_{p}$. We note that the combination of pathway-guided masking and ESM2-based initialization endows this linear projection with strong robustness against potential dominance by highly expressed genes: Weight distribution uniformity analysis (table S1) and spike-in perturbation tests (table S2) confirm that prototype tokens remain stable even under extreme expression perturbations. After passing through *m* prototypes simultaneously, the result is a set of prototype tokens $\left(v_{1},\ldots ,v_{m}\right)$ representing biologically relevant functions.

### scProtoTransformer architecture

The scProtoTransformer architecture models the interactions between the learned prototype tokens using six transformer layers with Flash Attention (*65*).

Once the prototype tokens are generated, a special [CLS] token is prepended to the sequence of prototype tokens. This token will eventually represent the embedding of the entire cell. The sequence, consisting of the [CLS] token followed by the prototype tokens, is then input into six stacked transformer layers.

The transformer layers in scProtoTransformer use Flash Attention, an optimized version of the attention mechanism that reduces memory usage and increases computation speed. Flash Attention performs the self-attention operation with reduced memory overhead by using memory-efficient techniques such as out-of-place operations. This enables the model to scale efficiently, even with large input sequences.

After passing through the six transformer layers, the final output is a sequence of embeddings, with the [CLS] token containing the most informative representation of the cell. This [CLS] token embedding is used as the cell embedding, which represents the biological state of the entire cell based on the learned prototype tokens and their interactions.

### Foundation model distillation

We designed a KD strategy guided by a foundation model for scProtoTransformer. The purpose of establishing this strategy is to enable scProtoTransformer to acquire the knowledge that the foundation model gains at a huge training cost, without the need for large-scale pretraining. Specifically, we use a frozen foundation model (such as scGPT) as a navigator and supervise the learning of scProtoTransformer through the cell embedding of the navigator. Different from the unsupervised pretraining of previous foundation models, we can dynamically use the labels in the data. When there are available labels in the data, we leverage the discriminative of the labels themselves to enhance the biological representation capability of scProtoTransformer. To train the model on data of limited scale, we use dynamic SFT (*66*), which can adaptively control gradients to prevent the model from overfitting. The training objective consists of KD loss and dynamic SFT. We first use KD loss on unlabeled data and then use dynamic SFT on labeled data.

#### KD loss

We use the foundation model scGPT as the teacher and scProtoTransformer as the student. We freeze the teacher and update the direction of the student’s output embedding based on cosine similarity. The cell embedding output by the teacher model is $c_{t}\in {\mathbb{R}}^{h}$, and the cell embedding output by the student model is $c_{r}\in {\mathbb{R}}^{h}$. First, we perform L2 normalization on both embeddings to eliminate the vector modulus and retain only the direction

$${\hat{c}}_{t}=\frac{c_{t}}{{\left\|c_{t}\right\|}_{2}},{\hat{c}}_{r}=\frac{c_{r}}{{\left\|c_{r}\right\|}_{2}}$$

Then, we define the KD loss using cosine similarity

$$L_{\text{KD}}=\frac{1}{N}\sum_{i=1}^{N}\left[1-{\hat{c}}_{t}^{\left(i\right)}\cdot {\hat{c}}_{r}^{\left(i\right)}\right]$$

where *N* is the total number of cells in the batch. By maximizing the similarity between the teacher and student embeddings of the same cell, the student model is able to quickly align the knowledge acquired by the foundation model during the large-scale pretraining phase.

#### Dynamic SFT

After scProtoTransformer is aligned using the KD loss, we use dynamic SFT to enhance the model’s discriminability. Standard SFT uses the cross-entropy loss. Dynamic SFT is a reinforcement learning–based SFT that avoids exploding gradients and prevents catastrophic forgetting caused by SFT. Let the model be the policy $\pi_{\theta }$, θ be the model parameters, the input sample be *x*, the ground-truth label be $y^{*}$, and the model’s predicted label be *y*. For distribution *D*, the expectation of the SFT loss is

$$L_{\text{SFT}}\left(\theta \right)=E_{\left(x,y^{*}\right)∼D}\left[-\text{log}\;\pi_{\theta }\left(y^{*}|x\right)\right]$$

The gradient of θ is

$$\nabla_{\theta }L_{\text{SFT}}=E_{\left(x,y^{*}\right)∼D}\left[-\nabla_{\theta }\text{log}\pi_{\theta }\left(y^{*}|x\right)\right]$$

Through importance sampling, the SFT gradient is written as the policy gradient form used in reinforcement learning

$$\nabla_{\theta }L_{\text{SFT}}=E_{x∼D_{x},y∼\pi_{\theta }\left(\cdot |x\right)}\left\{\frac{1\left[y=y^{*}\right]}{\pi_{\theta }\left(y|x\right)}\cdot \left[-\nabla_{\theta }\text{log}\pi_{\theta }\left(y|x\right)\right]\right\}$$

The above form exposes a key issue with SFT: When the model’s initial probability of the correct answer $\pi_{\theta }\left(y^{*}|x\right)$ is low, the importance weight $1/\pi_{\theta }\left(y^{*}|x\right)$ can become very large, leading to exploding gradients and unstable training. To eliminate the unstable weight $1/\pi_{\theta }\left(y^{*}|x\right)$, we directly multiply the raw SFT gradient by a stop-gradient probability $\text{sg}\left[\pi_{\theta }\left(y^{*}|x\right)\right]$ to offset it. Therefore, the dynamic SFT is

$$L_{\text{DSFT}}=E_{\left(x,y^{*}\right)∼D}\left\{-\text{sg}\left[\pi_{\theta }\left(y^{*}|x\right)\right]\cdot \text{log}\;\pi_{\theta }\left(y^{*}|x\right)\right\}$$

For a detailed derivation of the policy gradient, please refer to the Supplementary Materials.

### Scalable reference mapping

The scProtoTransformer framework supports scalable reference mapping at three biological levels: molecular, cellular, and donor levels.

#### Molecular level

The gene embeddings learned by scProtoTransformer can not only effectively capture the latent biological information of genes but also accurately assign labels to genes through supervised classification methods. This approach offers high flexibility, allowing extension to different cell types or biological conditions, and is suitable for various gene function prediction tasks.

In molecular-level reference mapping, we leverage the gene vocabulary within scProtoTransformer to extract the embedding representation of each gene. Specifically, the gene vocabulary contains embedding vectors of multiple genes, which are learned through the scProtoTransformer model. To perform effective reference mapping on these gene embeddings, we adopt two common supervised learning methods: LR and RF. These methods can learn the mapping relationship between genes and known labels in the gene embedding space. First, we construct an embedding representation for each gene. Then, we use known gene label data (such as the functional category of a gene or its expression status under certain biological conditions) to train a classifier. After training, the classifier can perform inference on unlabeled query genes and output predicted labels. These predicted labels help us accurately map the query genes to corresponding biological categories or functional groups, thereby achieving effective annotation of gene functions.

#### Cell level

When a single cell is input, scProtoTransformer can output a cell embedding. A cell classifier is used to assign predicted labels to each cell. We ensure that cell type annotations are consistent with the cell ontology (*67*, *68*), enabling the classifier to predict the cell type for any input cell. If we want the model to be more adaptable to a specific dataset, we can fine-tune scProtoTransformer on that particular dataset.

scProtoTransformer accepts gene expression data as input. When dealing with scATAC-seq data, we use Seurat (*34*) to convert the peaks of scATAC-seq into genes corresponding to scRNA-seq. For single-modal data, scProtoTransformer has a built-in batch integration function, we only need to infer cell embeddings to obtain a cross-batch joint space. For multimodal data, we first infer cell embeddings from the single-modal data, and then use an embedding alignment strategy (*69*) to integrate the cell embeddings across different modalities.

#### Donor level

In the donor-level reference mapping, we use an MIL module (*70*) to aggregate cell embeddings into a single donor-level embedding. In MIL, a set of cells from a single donor forms a bag, whereas each individual cell is treated as an instance. Typically, the bag label is known (the donor label), whereas the instance labels are unavailable. The goal is to learn a model that aggregates cell embeddings within each bag to form a donor-level embedding and uses this embedding for donor label prediction.

For this task, cell embeddings are derived from the scProtoTransformer model, which represents each cell in a high-dimensional feature space. Given a donor with *N* cells, the embedding of the *i*th cell is denoted as $e_{i}$, where i=1,2,…,N. The objective is to learn an aggregation function that combines these cell embeddings into a single donor embedding $e_{\text{donor}}$ and to predict the donor’s label based on this embedding.

We adopt an attention-based MIL approach. Specifically, we assign an attention score $\alpha_{i}$ to each cell embedding $e_{i}$, which reflects how relevant that cell is for the donor label. The attention scores are computed via a dense layer, where higher scores indicate a stronger correlation with the donor label.

Next, we aggregate the cell embeddings to form a donor-level embedding $e_{\text{donor}}$. The aggregation is performed as a weighted sum of the individual cell embeddings, where the weight for each cell embedding is its corresponding attention score $\alpha_{i}$. The aggregation function is defined as

$$e_{\text{donor}}=\sum_{i=1}^{N}\alpha_{i}e_{i}$$

This weighted sum ensures that cells with higher attention scores contribute more to the final donor-level embedding $e_{\text{donor}}$. Once we have the aggregated donor embedding $e_{\text{donor}}$, we can use a classifier to predict the donor’s label. In addition to label prediction, we can also backtrack to the instances that contribute most to the donor label prediction. Because attention scores $\alpha_{i}$ indicate the relevance of each cell to the donor label, higher attention scores correspond to cells that are more important for the prediction.

### Experimental setting

The knowledge database required by scProtoTransformer when generating functional patterns is Reactome (*71*). Specifically, we use the Reactome human pathway gene sets in GMT format obtained from the Reactome official website. For a given reference dataset, we extract the gene name list (20,009 genes in our implementation) and use GSEApy (*72*) to obtain *m* = 300 matched pathways (plus one fully connected catch-all node to retain uncovered genes). The number of genes per pathway ranges from 20 to 273, with a median of ∼56 and a mean of 78. We conducted a systematic sensitivity analysis of *m* (tables S3 and S4). The results show that model performance remains highly stable across *m* = 200 to 500, whereas performance degrades when *m* is too small (*m* = 50, where only the largest pathways are retained, leading to high interpathway redundancy and insufficient functional resolution) or when all available pathways are used (*m* = 655, where small annotation-sparse pathways introduce noise). The default *m* = 300 sits at the center of the stable range, balancing functional resolution against pathway redundancy. Preprocessing and differential analysis tool is scanpy (*73*). scProtoTransformer is trained using an NVIDIA RTX A6000 with 48-GB memory. Adam (*74*) optimizer with 1 × 10^−4^ learning rate is used to update model parameters. The batch size is set to 16.

We sampled 2 million cells from the human subset of scBaseCamp (*26*) and searched annotation information for each cell using SCimilarity (*68*), filtering out low-confidence labels. scProtoTransformer first aligned the cell embeddings output by scGPT across all cells. We then performed dynamic SFT on the model using the labeled data.
