# Supplementary material for: scProtoTransformer: Scalable reference mapping across molecules, cells, and donors
Source: Sci Adv. 2026 Aug 28;12(35):eaef0286. doi: 10.1126/sciadv.aef0286 (PMC13524047; doi:10.1126/sciadv.aef0286)
Supplement: Supplementary file 1 [file sciadv.aef0286_sm.pdf]

Science Advances
AAAS

Supplementary Materials for

scProtoTransformer: Scalable reference mapping across molecules, cells, and donors

Zhenchao Tang *et al.*

Corresponding author: Guanxing Chen, guanxing.chen@cityu-dg.edu.cn; Linlin You, youllin@mail.sysu.edu.cn; Calvin Yu-Chian Chen, cy@pku.edu.cn

Sci. Adv. **12**, eaef0286 (2026)
DOI: 10.1126/sciadv.aef0286

This PDF file includes:

Supplementary Text
Figs. S1 to S3
Tables S1 to S4

Supplementary Text

The standard supervised fine-tuning (SFT) objective minimizes the negative log-likelihood of expert demonstrations:

$$L_{\text{SFT}}(\theta) = \mathbb{E}_{(x,y^*)\sim\mathcal{D}} \left[-\log \pi_\theta(y^*|x)\right] \tag{S1}$$

where x is input data. y^* is expert label. $\pi_\theta(y^*|x)$ is model's likelihood of expert label given parameters θ. The gradient of this objective is:

$$\nabla_\theta L_{\text{SFT}}(\theta) = \mathbb{E}_{(x,y^*)\sim\mathcal{D}} \left[-\nabla_\theta \log \pi_\theta(y^*|x)\right] \tag{S2}$$

The reinforcement learning objective maximizes expected reward:

$$J(\theta) = \mathbb{E}_{x\sim\mathcal{D}_x, y\sim\pi_\theta(\cdot|x)} \left[r(x, y)\right] \tag{S3}$$

with the policy gradient theorem yielding:

$$\nabla_\theta J(\theta) = \mathbb{E}_{x\sim\mathcal{D}_x, y\sim\pi_\theta(\cdot|x)} \left[\nabla_\theta \log \pi_\theta(y|x) \cdot r(x, y)\right] \tag{S4}$$

Applying importance sampling to the SFT gradient:

$$\nabla_\theta L_{\text{SFT}}(\theta) = \mathbb{E}_{x\sim\mathcal{D}_x}\mathbb{E}_{y\sim\pi_\theta(\cdot|x)} \left[\frac{\delta(y, y^*)}{\pi_\theta(y|x)} \left(-\nabla_\theta \log \pi_\theta(y|x)\right)\right] \tag{S5}$$

where $\delta(y, y^*)$ is the Kronecker delta function (1 when $y = y^*$, 0 otherwise).

Defining implicit reward and importance weight functions:

$$r_{\text{SFT}}(x, y) = \delta(y, y^*),\ w(y|x) = \pi_\theta(y|x)^{-1} \tag{S6}$$

yields the RL-equivalent form:

$$\nabla_\theta L_{\text{SFT}}(\theta) = -\mathbb{E}_{x\sim\mathcal{D}_x, y\sim\pi_\theta(\cdot|x)} \left[w(y|x) r_{\text{SFT}}(x, y) \nabla_\theta \log \pi_\theta(y|x)\right] \tag{S7}$$

The importance weight $w(y|x) = \pi_\theta(y^*|x)^{-1}$ introduces instability:

- Gradient explosion when $\pi_\theta(y^*|x) \to 0^+$.

- High-variance updates during early training phases.
- Degraded generalization due to overemphasis on low-likelihood labels.

Dynamic SFT (DFT) introduces a stabilizing coefficient $\pi_\theta(y^*|x)$:

$$\nabla_\theta L_{\mathrm{DFT}}(\theta) = \mathbb{E}_{(x,y^*)\sim\mathcal{D}}\left[-\pi_\theta(y^*|x)\nabla_\theta \log \pi_\theta(y^*|x)\right] \tag{S8}$$

where $\pi_\theta(y^*|x)$ is treated as a fixed scaling factor (stop-gradient).

The corresponding DFT loss function:

$$L_{\mathrm{DFT}}(\theta) = \mathbb{E}_{(x,y^*)\sim\mathcal{D}}\left[-\pi_\theta(y^*|x)\log \pi_\theta(y^*|x)\right] \tag{S9}$$

Supplementary Figures and Tables

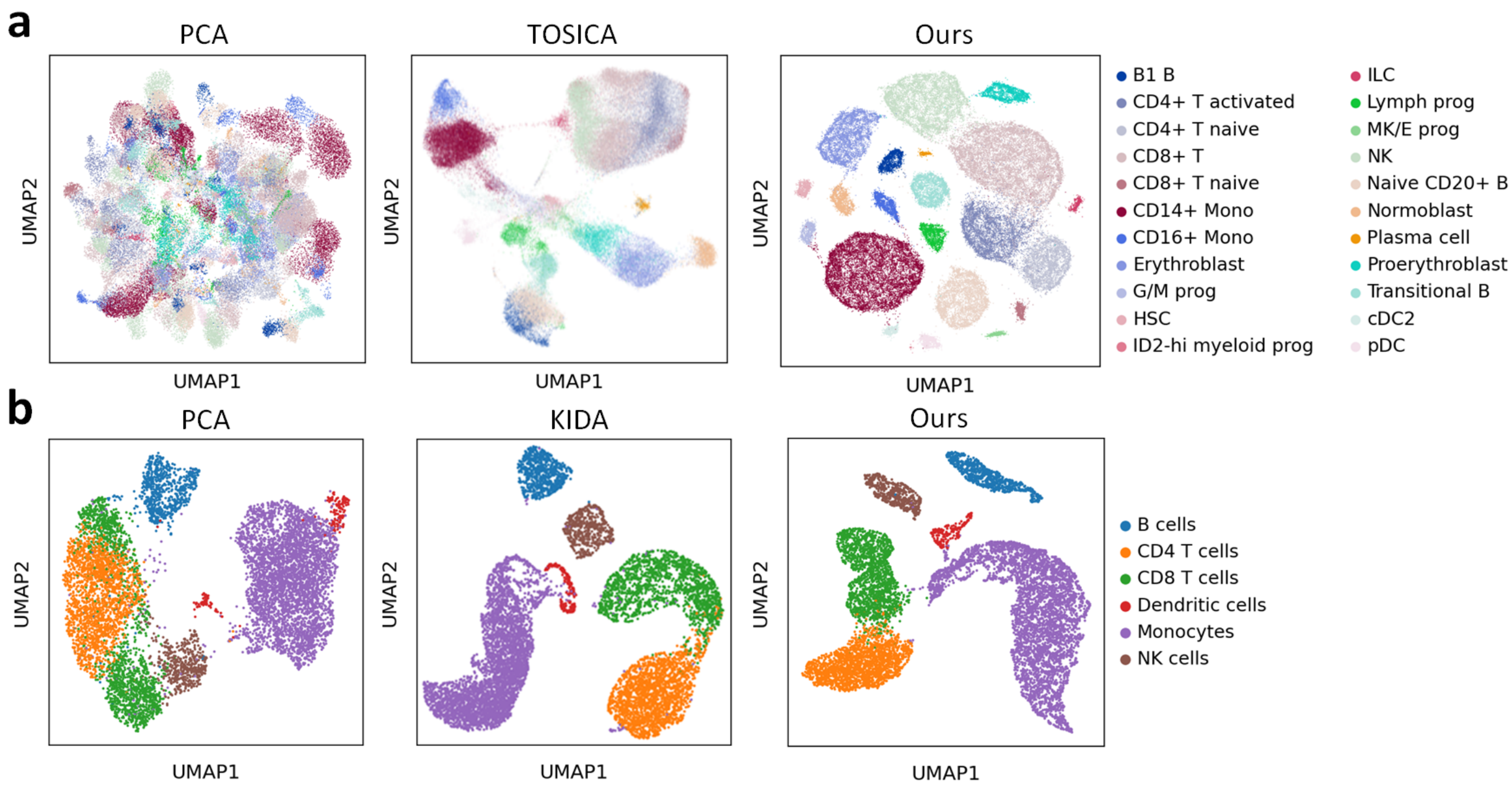

Figure S1: Cell-scale UMAP visualization. Figure a: Cell embedding UMAP of the BMMC dataset (measured using scRNA-seq). From left to right, PCA, TOSICA (a method specific to scRNA-seq), and our cell embeddings are visualized, colored by cell type. Figure b: Cell embedding UMAP of the PBMC dataset (measured using scATAC-seq). From left to right, PCA, KIDA (a method specific to scATAC-seq), and our cell embeddings are visualized, colored by cell type.

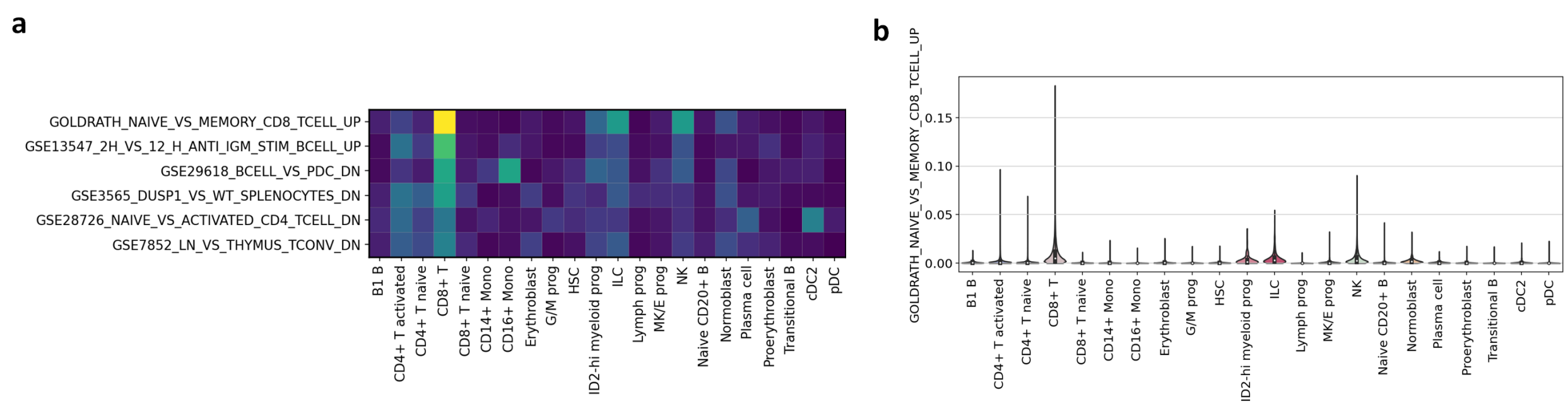

Figure S2: Results of interpretable attention on prototypes. Figure a: The vertical axis represents the top six prototypes specific to CD8+ T cells, and the horizontal axis represents different cell types, with attention visualized using a heatmap. Figure b: For a given prototype (the first prototype in Figure a, named by pathway), the gene expression distribution corresponding to the prototype is calculated across different cell types.

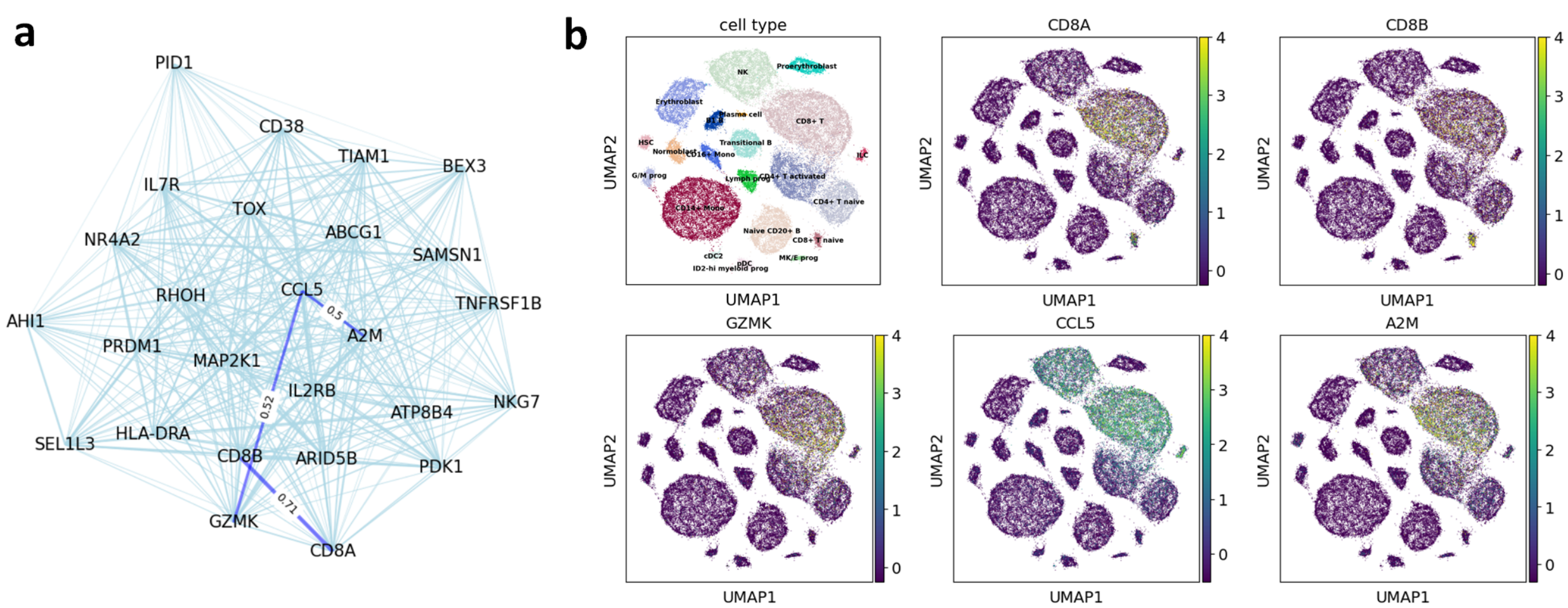

Figure S3: Gene interpretability. Figure a: The CD8+ T-specific gene co-expression network from scProtoTransformer. The edges with high similarity values are shown in dark blue. Figure b: Validation results of important genes based on UMAP visualization.

Table S1: Weight distribution uniformity analysis of prototype tokens across 20 representative Reactome pathways, comparing ESM2 initialization versus random initialization. For each pathway, we compute the L2 norm of each gene's row in the trained prototype tokenizer weight matrix to obtain a per-gene weight, then normalize these weights to sum to one. Entropy measures how uniformly these normalized weights are spread across genes within a pathway (1.0 = perfectly uniform), reflecting whether all member genes contribute equally to the prototype token. Gini coefficient quantifies inequality among the gene weights (0.0 = perfectly equal), with higher values indicating that a few genes dominate the prototype representation. The 20 pathways were selected to span diverse biological functions (immune regulation, metabolism, signal transduction, cell cycle, apoptosis, and development) and a range of pathway sizes (from 21 to 249 genes).

**Pathway**	**Genes**	**Entropy (ESM2)**	**Entropy (Rand)**	**Gini (ESM2)**	**Gini (Rand)**
Interferon Sig.	142	0.968	0.722	0.094	0.371
Cell Cycle Checkpt.	110	0.985	0.804	0.058	0.273
Platelet Cytosolic	80	0.957	0.715	0.108	0.368
Innate Immune	234	0.991	0.826	0.043	0.249
Signal by ILs	105	0.973	0.736	0.087	0.356
Metab. of Amino Acids	187	0.989	0.795	0.062	0.291
Axon Guidance	238	0.994	0.823	0.045	0.252
Class I MHC	230	0.987	0.799	0.058	0.282
Cytokine Signal.	249	0.992	0.831	0.044	0.241
Signal Transd.	89	0.965	0.717	0.099	0.385
Gene Expression	101	0.978	0.751	0.074	0.342
mRNA Processing	149	0.984	0.773	0.066	0.318
Cell Cell Comm.	110	0.971	0.728	0.086	0.371
Apoptosis	56	0.948	0.695	0.121	0.418
Transcription	175	0.986	0.781	0.063	0.305
Membrane Traffic.	123	0.979	0.764	0.072	0.328
Metab. of Carbohy.	229	0.990	0.819	0.046	0.258
ERK/MAPK Targets	21	0.921	0.654	0.143	0.452
PI3K/AKT Activ.	36	0.939	0.683	0.128	0.426
NF-κB in B Cells	61	0.951	0.706	0.115	0.401
**Mean**	—	**0.972**	**0.756**	**0.081**	**0.335**

Table S2: Spike-in perturbation robustness test. Cosine similarity and L2 distance between original and perturbed prototype tokens under different spike-in magnitudes in raw count space, comparing ESM2 initialization versus random initialization. For each pathway, the spike-in gene is selected as the gene with the highest mean expression across all cells in the reference dataset within that pathway, representing the worst-case scenario for single-gene dominance. Note that the effective input perturbation grows sub-linearly with the spike-in magnitude due to log1p preprocessing (e.g., a 100× raw-count amplification corresponds to roughly 3× in the log-transformed model input), which explains the observed saturating behavior at larger magnitudes. Results are averaged over the same 20 representative Reactome pathways used in Table S1.

**Spike-in Magnitude**	**Cos Sim (ESM2)**	**Cos Sim (Random)**	**L2 Dist (ESM2)**	**L2 Dist (Random)**
0x (baseline)	1.000	1.000	0.000	0.000
2x	0.992	0.913	0.097	0.341
5x	0.978	0.831	0.214	0.563
10x	0.961	0.738	0.336	0.812
20x	0.945	0.636	0.451	1.087
50x	0.926	0.524	0.571	1.345
100x	0.911	0.447	0.642	1.521

Table S3: Hyperparameter m sensitivity analysis: performance across all tasks. Performance of scProto-Transformer under different numbers of prototypes (m). The default m=300 is highlighted in bold.

m	TF range	Dosage sens.	Biv. vs no-me.	Biv. vs Lys4	BMMC	PBMC	Pan-cancer	Kang (mean)	Kang (attn)
50	0.55	0.87	0.91	0.89	0.91	0.95	0.88	0.77	0.79
100	0.58	0.90	0.93	0.92	0.93	0.96	0.90	0.79	0.80
200	0.61	0.92	0.96	0.93	0.94	0.98	0.90	0.79	0.80
**300**	**0.62**	**0.93**	**0.95**	**0.94**	**0.94**	**0.97**	**0.91**	**0.81**	**0.83**
400	0.64	0.92	0.95	0.94	0.94	0.97	0.91	0.80	0.82
500	0.62	0.92	0.93	0.95	0.93	0.96	0.90	0.79	0.81
655[†]	0.62	0.93	0.94	0.95	0.92	0.96	0.91	0.80	0.81

[†]All available Reactome pathways after filtering (used for $m \geq 655$).

Table S4: Hyperparameter m sensitivity analysis: auxiliary metrics. The m pathways are selected by ranking all Reactome pathways by the number of overlapping genes with the reference gene list and retaining the top m. Gene coverage is the proportion of the 20,009 selected genes covered by at least one of the m pathways. Jaccard redundancy is the mean pairwise Jaccard index across all pathway pairs. Avg. Genes/Pathway is the mean number of genes per selected pathway.

m	**Gene Coverage**	**Jaccard Redundancy**	**Avg. Genes/Pathway**
50	18.3%	0.040	160
100	21.1%	0.039	124
200	23.4%	0.034	95
**300**	**24.3%**	**0.023**	**78**
400	24.4%	0.016	65
500	24.5%	0.013	56
655†	24.7%	0.009	47

†All available Reactome pathways after filtering (used for $m \geq 655$).
